# miR-24-3p/KLF8 Signaling Axis Contributes to LUAD Metastasis by Regulating EMT

**DOI:** 10.1155/2020/4036047

**Published:** 2020-04-28

**Authors:** Pengyu Jing, Nianlin Xie, Nan Zhao, Ximing Zhu, Pei Li, Guizhou Gao, Haizhou Dang, Zhongping Gu

**Affiliations:** ^1^Department of Thoracic Surgery, The Second Affiliated Hospital, Air Force Medical University, Xi'an 710038, China; ^2^Department of Public Health, Xi'an Medical University, Xi'an 710021, China

## Abstract

Reprogramming of the tumor immune microenvironment is a salient feature during metastasis in LUAD. miR-24-3p and KLF8, which are key regulators of the tumor immune microenvironment, had been proved to show metastasis-promoting property in LUAD. However, whether miR-24-3p could regulate LUAD metastasis by targeting KLF8 remains unclear. This study explored the functions and mechanisms of miR-24-3p/KLF8 signaling in advanced LUAD. The expression level of miR-24-3p and KLF8 were tested in LUAD patients, and the corelation of miR-24-3p and KLF8 was evaluated. The interaction of miR-24-3p and KLF8 was demonstrated by luciferase reporter activity assay, *in vitro* migration and invasion studies, and *in vivo* metastatic studies. miR-24-3p level was downregulated in LUAD and negatively associated with KLF8 mRNA expression. miR-24-3p controls LUAD metastasis by directly targeting KLF8 and inducing Snail and E-cadherin expressions. Targeting the miR-24-3p/KLF8/EMT axis might be of great therapeutic value to advanced LUAD patients.

## 1. Introduction

Lung cancer ranks one of the most fearsome malignancies globally due to the foremost cause of mortality worldwide [1, 2]. Lung adenocarcinoma (LUAD) contributes to the major histologic type of lung cancer with an unfavorable 5-year survival rate of only 15% [3–5]. Metastasis is the leading cause of cancer-related death for advanced LUAD [1, 2, 4–6]. However, a full understanding of the underlying mechanisms controlling LUAD metastasis is still largely insufficient. Mounting evidences have confirmed that reprogramming the tumor immune microenvironment is a major process that drives LUAD metastasis by EMT activation and provided multiple targetable checkpoint molecules for advanced LUAD [7, 8]. The dysregulation of microRNAs (miRNAs) is broadly participated in the pathogenesis of LUAD and functionally act as a key contributor to cancer cell metastasis [9, 10]. Yet, the key dysregulated miRNAs and the exact mechanisms in LUAD metastasis remain unrevealed. miR-24-3p is one of the most frequently dysregulated EMT-associated miRNAs in carcinogenesis, which is best known for its role in regulating cancer cell metastasis, including human breast adenocarcinoma, hepatocellular carcinoma, gastric cancer, prostate carcinoma, and lung cancer [11–15]. Importantly, miR-24-3p has been reported to be involved in LUAD metastasis by targeting fibroblast growth factor receptor 3 (FGFR3), inhibitor of growth 5 (ING5), and sex-determining region Y-box 7(SOX7) [11, 15, 16], indicating that miR-24-3p plays a pivotal role in LUAD metastasis. Despite the significant role of miR-24-3p in metastatic LUAD, the potential mechanisms of miR-24-3p in regulating cell invasion and migration of LUAD are not fully clear yet.

Krüppel-like transcription factor 8 (KLF8) belongs to the Krüppel-like C2H2 zinc-finger transcription factor family, which is involved in multiple cellular biological processes, such as cell proliferation, differentiation, and migration [17, 18]. KLF8 has been reported to maintain the invasive capacity of cancer cells by inducing epithelial-to-mesenchymal transition (EMT) [17–19]. Of importance, KLF8 has been implicated to be regulated by miRNAs in metastatic progression of lung cancer. miR-1236-3p and miR-135a could inhibit the metastatic procedure of lung cancer by targeting KLF8 [20, 21]. However, whether miR-24-3p could regulate LUAD metastasis by targeting KLF8 remains unclear.

This study demonstrated that miR-24-3p level was downregulated in LUAD and negatively associated with KLF8 mRNA expression. miR-24-3p controls LUAD metastasis by directly targeting KLF8 and inducing EMT activation. Targeting the miR-24-3p/KLF8/EMT axis might be of great therapeutic value to advanced LUAD patients.

## 2. Materials and Methods

### 2.1. Tissues, Cell Lines, and Reagents

Surgical specimens, which contain 18 pairs of tumor tissues and nontumor tissues of LUAD, were collected from Tangdu Hospital according to the Medical Ethics Committee's guidelines. Tissue RNA was extracted for real-time PCR evaluation, and tissue protein was collected and analyzed by Western blot. A549 and H1299 human cell lines of LUAD were ordered from the Cell bank of Chinese Academy of Sciences (Shanghai, China) after being tested for mycoplasma contamination. All cells were cultured in complete DMED (plus 10% FBS and 100 U/ml penicillin sodium and 100 *μ*g/ml streptomycin) in 37°C incubator with 5% CO_2_. Primary antibodies used in Western blot study were as follows: KLF8 (Abcam, ab168527, 1: 1000), Snail (Cell Signaling Technology, #3879, 1: 1000), E-cadherin (Cell Signaling Technology, #3195, 1: 1000), and *β*-actin (Cell Signaling Technology, #3700, 1: 5000). HRP-conjugated secondary antibodies and the HRP substrate kit were ordered from the Merck Company. Lipofectamine™ was purchased from Invitrogen Company.

### 2.2. RNA Extraction and qRT–PCR

RNA isolation was carried out by using TRIzol (Life Technologies) according to previous reports [11]. Total 3 *μ*g/RT reaction of RNA from the LUAD cells or tissues was used for analysis of the mRNA level of the target gene by real-time quantitative PCR on the Light Cycler 480II (Roche). The primer sequences information is listed as follows: miR-24-3p F: TGGCTCACATCAGCAGGAACA; U6 F: GGAACGATACAGAGAAGATTAGC, U6 R: TGGAACGCTTCACGAATTTGCG; KLF8 F: GCTCACCGCAGAATCCATACA, KLF8 R: GTGCACCGAAAAGGCTTGAT; and *β*-actin F: CATGTACGTTGCTATCCAGGC, *β*-actin R: CTCCTTAATGTCACGCACGAT.

### 2.3. Protein Collection and Western Blots

Proteins of cells were extracted with a lysis buffer (containing RIPA, protease inhibitors, and phosphatase inhibitors). 20 *μ*g of each protein was used for SDS-PAGE. Then, the proteins were transferred onto membranes and incubated with primary antibodies overnight. After incubation with HRP-conjugated secondary antibodies, membranes were visualized using Immobilon™ Western.

### 2.4. Luciferase Reporter Activity Assay

Plasmids containing the wild-type (WT) KLF8-3′-UTR sequence and a mutant KLF8-3′-UTR sequence were synthesized by GeneCopoeia (Shanghai, China). Cells were transfected with the plasmids and harvested and lysed for luciferase assay by using a Dual Luciferase Assay kit (GeneCopoeia) according to the manufacturer's instructions as described previously [11, 22].

### 2.5. *In Vitro* Functional Studies

*In vitro* migration and invasion assays were performed by using transwell chambers according to the manufacturer's instructions. For migration assay, 5^∗^10^4^ cells were seeded in a serum-free medium in the upper chamber. For invasion assay, the chambers were covered with Matrigel previously and dried overnight. 1^∗^10^5^ cells were seeded in DMEM with 1% FBS in the upper chamber. A medium supplemented with 20% FBS was added into the lower chamber. Cells remaining on the upper membrane after 24-hour incubation were removed, and cells on the lower surface of the membrane were fixed, stained, and counted. For wound healing assay, cells were seeded in 6-well plates until reaching confluence. Then, wounds were scratched by using sterile tips, and wound closure was recorded every 12 hours by using a microscope.

### 2.6. In Vivo Animal Experiments

All of the mouse studies were approved by Animal Care and Use Committee of the Ethics Committee of Tangdu Hospital. Nude mice (BALB/C strain, 6-week old, male) were used as the metastatic model. 3^∗^10^6^ cells were injected through the tail veins for 5 weeks. D-luciferin (Roche) was injected intraperitoneally in the mice at week 2 and week 5, and the bioluminescence images were gained with an IVIS 100 Imaging System (Xenogen).

## 3. Statistical Analysis

All statistical analyses for this study were assessed by using SPSS 20.0 software (SPSS Inc., USA). Student's *t*-test was used to evaluate the quantitative data between groups. Kaplan-Meier analyses, as well as the log-rank test, were used to estimate the overall survival. Pearson's correlation coefficients were used to evaluate the correlation between two indices in clinical samples. A difference at *p* < 0.05 was considered statistically significant.

## 4. Results

### 4.1. miR-24-3p Was Negatively Associated with KLF8 mRNA Expression and Was Closely Related to EMT Markers in LUAD

**Figure 1:**
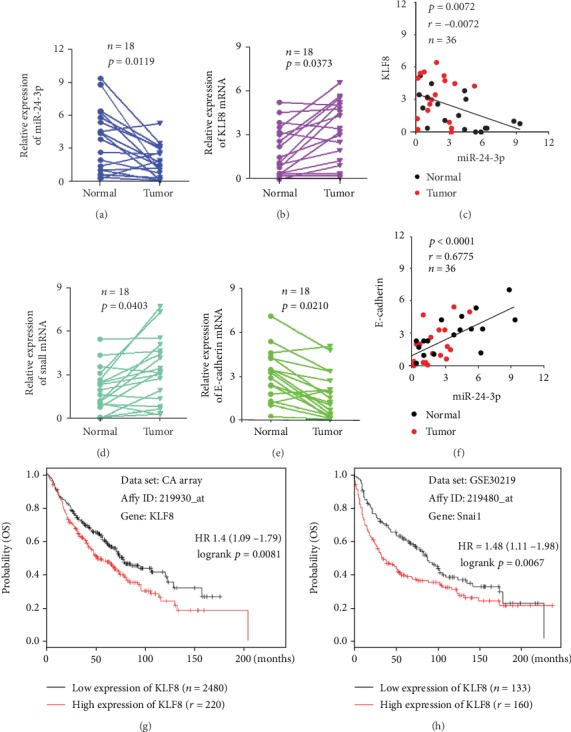
miR-24-3p was negatively associated with KLF8 mRNA expression and was closely related to EMT markers (Snail and E-cadherin mRNA). (a) miR-24-3p expression in lung adenocarcinoma was less than that in normal tissues. (b) KLF8 mRNA expression in lung adenocarcinoma was higher than that in normal tissues. (c) Regression analysis comparing miR-24-3p and KLF8 mRNA expressions in lung adenocarcinoma tissues and the corresponding normal tissues. (d) Snail mRNA expression in lung adenocarcinoma tissues was higher than that in normal tissues. (e) E-cadherin mRNA expression in lung adenocarcinoma was less than that in normal tissues. (f) Regression analysis comparing miR-24-3p and E-cadherin mRNA expressions in lung adenocarcinoma tissues and the corresponding normal tissues. (g, h) Kaplan-Meier estimates of the cumulative survival rate based on the Kaplan-Meier plotter database. The higher KLF8 and Snail mRNA expression were closely related to poor prognosis in lung adenocarcinoma.

We initially analyzed the expression of miR-24-3p and KLF8 in 18 pairs of tumor tissues and nontumor tissues of LUAD by qRT–PCR and found that the expression levels of miR-24-3p declined in LUAD tissues than those in adjacent nontumor tissues (Figure 1(a)), while KLF8 mRNA expression was increased in LUAD tissues than that in adjacent nontumor tissues (Figure 1(b)). What is more, we found that miR-24-3p expression level was negatively associated with KLF8 mRNA expression level (Figure 1(c)). Together, these data indicated that miR-24-3p involved in LUAD progression may be associated with KLF8. To further confirm whether the dysregulation of miR-24-3p in LUAD was associated with EMT, we analyzed the mRNA levels of key EMT markers, Snail and E-cadherin, and revealed that the Snail mRNA level was increased, while the E-cadherin mRNA level was decreased in LUAD (Figures 1(d) and 1(e)). In addition, regression analysis comparing miR-24-3p and E-cadherin mRNA expressions revealed that miR-24-3p levels were closely associated with E-cadherin (Figure 1(f)), suggesting that miR-24-3p involved in LUAD progression may be associated with EMT. Further analysis by Kaplan-Meier plotter database implicated that both KLF8 and Snail mRNA expression levels were closely related to poor clinical outcomes of LUAD, manifesting that KLF8 and Snail are critical in the progression of LUAD (Figures 1(g) and 1(h)).

### 4.2. miR-24-3p Regulates KLF8, Snail, and E-Cadherin Expressions and Directly Targets KLF8

**Figure 2:**
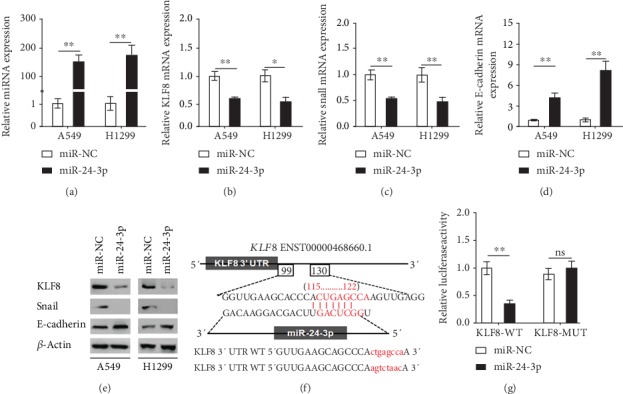
miR-24-3p regulated KLF8, Snail, and E-cadherin expressions in lung adenocarcinoma cells, and KLF8 was a direct target of miR-24-3p. (a) qRT-PCR analysis of miR-24-3p expression in A549 and H1299 cells transfected with either miR-NC or miR-24-3p mimics. (b–d) mRNA expression of KLF8 (b), Snail (c), and E-cadherin (d) in A549 and H1299 cells transfected with either miR-NC or miR-24-3p mimics was detected by qRT-PCR. (e) Expression of KLF8, Snail, and E-cadherin in A549 and H1299 cells transfected with either miR-NC or miR-24-3p mimics was detected by Western blot. (f) miR-24-3p binding sites in 3′-UTR of KLF8 were predicted by TargetScan. The seed region of miR-24-3p and the recognition site in the KLF8 3′-UTR are shown in red. The sequences of the WT and MUT KLF8 3′-UTR were used for the dual luciferase reporter construct. (g) The pMIR-REPORT vector and either KLF8-WT or KLF8-MUT plasmids were cotransfected with miR-24-3p or miR-NC mimics in H1299 cells, and the relative luciferase activity was detected. Data are mean ± SD. ^∗^*p* < 0.05, ^∗∗^*p* < 0.01.

To further confirm the regulator role of miR-24-3p for KLF8, Snail, and E-cadherin, we overexpressed miR-24-3p in A549 and H1299 cells (Figure 2(a)). KLF8 and Snail mRNA levels were significantly downregulated, while E-cadherin mRNA level was significantly upregulated when the cells overexpressed miR-24-3p (Figures 2(b)–2(d)). Western blot assay also showed that the protein levels were correspondingly changed after the cells overexpressed miR-24-3p (Figure 2(e)). The wild-type (WT) KLF8 3′-UTR sequence and a mutant KLF8 3′-UTR sequence were inserted into a luciferase reporter vector to further validate whether miR-24-3p regulated the expression of KLF8 by binding to its 3′-UTR (Figure 2(f)). As determined by luciferase activity assay, miR-24-3p conspicuously suppressed the relative luciferase activity in presence of WT reporter construct of KLF8 3′-UTR, while there was no significant differences of relative luciferase activity when the mutant-type reporter construct of KLF8 3′-UTR was constructed (Figure 2(g)), suggesting that miR-24-3p could directly target KLF8.

### 4.3. KLF8 Promoted LUAD Cell Metastasis *In Vitro* and *In Vivo*

**Figure 3:**
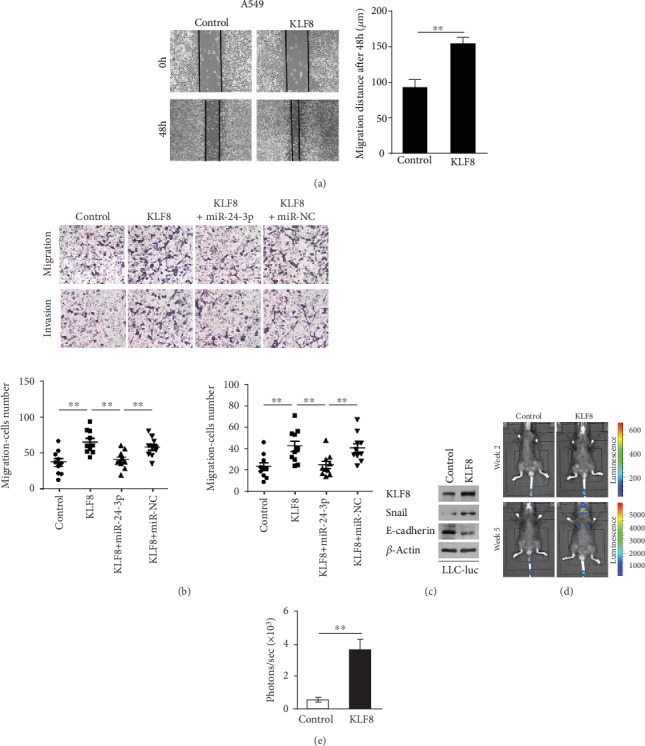
KLF8 promoted lung adenocarcinoma cell metastasis in vitro and in vivo. (a) The wound healing assay (*n* = 5) demonstrated that KLF8 overexpression strengthened the migration of A549 cell. (b) The migration and invasion potentials of the A549 cell as indicated were evaluated using transwell assay, and the numbers of migrated and invaded cells are shown (*n* = 10). (c) Protein expression of KLF8, Snail, and E-cadherin in LLC-luc-Control or LLC-luc-KLF8 cells was detected by Western blot. (d) LLC-luc-Control or LLC-luc-KLF8 cells were injected IV into C57BL/6 mice, and tumor growth and metastasis were monitored by in vivo imaging. (e) Intensities of luciferase signal in lungs of LLC-luc-KLF8 mice were higher than that in lungs of LLC-luc-Control mice. Data are mean ± SD. ^∗∗^*p* < 0.01.

The metastatic role of KLF8 in LUAD was further tested in A549 cells *in vitro* and *in vivo*. Wound healing assay showed that KLF8 overexpression strengthened the migration capacity of A549 cell after 48 hours of induction (Figure 3(a)). Consistently, transwell analysis demonstrated that KLF8 overexpression enhanced the migration and invasion ability in A549 cells. What is more, the enhanced migration and invasion capacity induced by KLF8 overexpression could significantly reverse after the cells cotransfected with miR-24-3p, suggesting that miR-24-3p could control LUAD metastasis at least partly depending on targeting of KLF8 (Figure 3(b)). As expected, KLF8 overexpression further lead to an increase of Snail protein level, while the E-cadherin protein level was suppressed after KLF8 overexpression, suggesting that KLF8 could regulate EMT in LUAD (Figure 3(c)). *In vivo* metastasis model showed that KLF overexpression increased the metastasis of A549 cells as evidenced by bioluminescence images (Figures 3(d) and 3(e)). All the above data confessed that KLF8 promoted LUAD cell metastasis.

### 4.4. miR-24-3p Regulated EMT through KLF8

**Figure 4:**
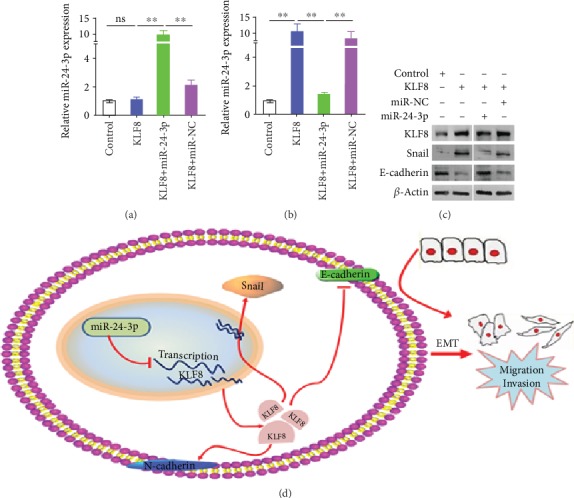
miR-24-3p regulated EMT through KLF8. (a, b) Expression of miR-24-3p (a) and KLF8 (b) mRNAs in the cell as indicated was detected by qPCR. Transfection of miR-24-3p mimics in A549 cells with KLF8 overexpression increased miR-24-3p expression and also decreased KLF8 mRNA expression. (c) Expression of KLF8, Snail, and E-cadherin in A549 cells with overexpression of KLF8, miR-24-3p, or their corresponding genes. (d) A schematic diagram for roles of miR-24-3p in lung adenocarcinoma and interactions with KLF8 and EMT programs. miR-24-3p suppressed KLF8 transcription directly, and the reduced KLF8 levels resulted in a decrease in Snail expression and an increase in E-cadherin expression, which in turn promoted EMT program and ultimately lead to enhanced metastasis of lung adenocarcinoma. Data are mean ± SD. ^∗∗^*p* < 0.01.

Further, we found that cotransfection of miR-24-3p mimics in A549 cells with KLF8 overexpression increased miR-24-3p expression and also decreased KLF8 mRNA expression as determined by qRT-PCR (Figures 4(a) and 4(b)). The protein expression of Snail and E-cadherin was also reversed when cotransfected with miR-24-3p mimics in A549 cells with KLF8 (Figure 4(c)). These results indicate that miR-24-3p could regulate EMT through KLF8, thus promoting LUAD metastasis.

## 5. Discussion

In this study, we found that miR-24-3p was frequently silenced in LUAD patients and controls LUAD metastasis by directly targeting KLF8 and inducing EMT, which broaden the understanding of the miR-24-3p/KLF8/EMT axis in the pathogenesis of LUAD and provided therapeutic indications to advanced LUAD patients.

It is well known that miRNAs, especially metastatic-related miRNAs, such as miR-1236-3p, miR-24-3p, and miR-135a, which usually regulates EMT-related genes to control metastasis, is often dysregulated during the pathogenesis of LUAD [21, 23]. The miR-24-3p expression level has been reported to be silenced in LUAD and contributed to LUAD metastasis by regulating multiple targets, including FGFR3, ING5, and SOX7 [11, 15, 16]. Therefore, miR-24-3p was selected to be the most researched miRNA in LUAD. In this study, we first studied the expression status of miR-24-3p in LUAD patients and presented that miR-24-3p showed a lower expression level in LUAD tissues than in adjacent nontumor tissues as previously described [11, 16, 24]. In addition, regression analysis revealed that miR-24-3p levels were closely associated with E-cadherin in LUAD, suggesting that miR-24-3p involved in LUAD progression may be associated with EMT. It is well known that microRNAs are known for its role in posttranscriptionally regulating target genes by binding to its 3′-UTR. Therefore, to confirm whether the miR-24-3p is an upstream regulator of KLF8, we overexpressed miR-24-3p in LUAD cell lines and explored the interaction of KLF8 and miR-24-3p in LUAD cells; using Western blot assay and luciferase reporter assay, we demonstrated that KLF8 was a direct target of miR-24-3p and that the downregulation of miR-24-3p could affect KLF8 protein expression in LUAD.

As a KLF family member, KLF8 is often aberrantly expressed in many types of cancer and its overexpression is significantly correlated with oncogenic transformation. Also, KLF8 is known for its ability of inducing EMT and drug resistance in tumor progression [25–28]. KLF8 has been proved to be regulated by miR-1236-3p and miR-135a in metastatic progression of lung cancer [20, 21]. Our study confirmed that KLF8 expression in LUAD was higher than that in nontumor tissues. Using the public prediction database, we found that KLF8 mRNA expression level was positively correlated to poor overall survival of LUAD. *In vitro* and *in vivo* functional studies also revealed that KLF8 promoted LUAD cell metastasis and invasion, manifesting that KLF8 is critical in the progression of LUAD. However, the role of miR-24-3p in regulating KLF8 expression is still unclear. Therefore, we wondered whether miR-24-3p is responsible for LUAD metastasis via interacting with KLF8. Here, we reported that miR-24-3p could directly target KLF8 by binding to its 3′-UTR, which contributed to further EMT in LUAD. Thus, the miR-24-3p/KLF8/EMT axis may be responsible for the aggressive behaviors of LUAD, paving the way for developing novel targeted drugs and designing new therapeutic strategy. As a kind of easy-to-metastasized cancer, tumor immune microenvironment played a significant role in LUAD by EMT activation [29]. EMT might contribute to immune escape through multiple routes. Our data suggested that the miR-24-3p/KLF8 axis is responsible for EMT activation, which provides new insights into immunotherapy focused on the miR-24-3p/KLF8 axis.

## 6. Conclusions

In conclusion, we found that miR-24-3p and KLF8 played an important role in EMT of LUAD. Importantly, we demonstrated that miR-24-3p could directly target KLF8. We believed that the currently identified miR-24-3p/KLF8/EMT axis could provide a novel insight into the molecular basis of advanced LUAD and represent potential therapeutic targets for the treatment of metastatic LUAD.

## Data Availability

The data used to support the findings of this study are available from the corresponding author upon request.

## Abbreviations

LUAD: Lung adenocarcinoma
miRNAs: MicroRNAs
BC: Breast cancer
FGFR3: Fibroblast growth factor receptor 3
SOX7: Sex-determining region Y-box 7
SOX18: Sex-determining region Y-box 18
KLF8: Krüppel-like transcription factor 8
EMT: Epithelial-to-mesenchymal transition
WT: Wild type.
